# The Evaluation on the Cadmium Net Concentration for Soil Ecosystems

**DOI:** 10.3390/ijerph14030297

**Published:** 2017-03-12

**Authors:** Yu Yao, Pei-Fang Wang, Chao Wang, Jun Hou, Ling-Zhan Miao

**Affiliations:** Key Laboratory of Integrated Regulation and Resource Development on Shallow Lakes, Ministry of Education, College of Environment, Hohai University, Nanjing 210098, China; yu2011358@163.com (Y.Y.); cwang@hhu.edu.cn (C.W.); hhuhjyhj@126.com (J.H.); mlz1988@126.com (L.-Z.M.)

**Keywords:** DGT, cadmium bioavailability, soil, Yixing, paddy, zizania aquatica

## Abstract

Yixing, known as the “City of Ceramics”, is facing a new dilemma: a raw material crisis. Cadmium (Cd) exists in extremely high concentrations in soil due to the considerable input of industrial wastewater into the soil ecosystem. The in situ technique of diffusive gradients in thin film (DGT), the ex situ static equilibrium approach (HAc, EDTA and CaCl_2_), and the dissolved concentration in soil solution, as well as microwave digestion, were applied to predict the Cd bioavailability of soil, aiming to provide a robust and accurate method for Cd bioavailability evaluation in Yixing. Moreover, the typical local cash crops—paddy and zizania aquatica—were selected for Cd accumulation, aiming to select the ideal plants with tolerance to the soil Cd contamination. The results indicated that the biomasses of the two applied plants were sufficiently sensitive to reflect the stark regional differences of different sampling sites. The zizania aquatica could effectively reduce the total Cd concentration, as indicated by the high accumulation coefficients. However, the fact that the zizania aquatica has extremely high transfer coefficients, and its stem, as the edible part, might accumulate large amounts of Cd, led to the conclusion that zizania aquatica was not an ideal cash crop in Yixing. Furthermore, the labile Cd concentrations which were obtained by the DGT technique and dissolved in the soil solution showed a significant correlation with the Cd concentrations of the biota accumulation. However, the *ex situ* methods and the microwave digestion-obtained Cd concentrations showed a poor correlation with the accumulated Cd concentration in plant tissue. Correspondingly, the multiple linear regression models were built for fundamental analysis of the performance of different methods available for Cd bioavailability evaluation. The correlation coefficients of DGT obtained by the improved multiple linear regression model have not significantly improved compared to the coefficients obtained by the simple linear regression model. The results revealed that DGT was a robust measurement, which could obtain the labile Cd concentrations independent of the physicochemical features’ variation in the soil ecosystem. Consequently, these findings provide stronger evidence that DGT is an effective and ideal tool for labile Cd evaluation in Yixing.

## 1. Introduction

Yixing, an ancient “City of Ceramics” and a modern industrially developed city, is facing a new dilemma: serious soil ecosystem contamination. Cadmium (Cd) is relatively rare in the Earth’s crust; however, due to electroplating, wastewater irrigation, mining, smelting and the abuse of pesticides and fertilizers, the Cd contamination in soils seems increasingly severe in Yixing [1,2,3]. Cd could cause serious nervous system damage to humans through bioaccumulation and bioamplification in members of the food chain, such as cereals and vegetables [2,3]. In view of this, Cd has been classified as a Group 1 human carcinogen by the International Agency for Research on Cancer (IARC) [2,3,4,5]. Due to its high mobility and widespread occurrence, it is of great urgency to monitor the Cd bioavailability in the soil ecosystem of Yixing.

At present, there exist numerous methods for Cd bioavailability evaluation: the microwave digestion, dissolved concentration in soil solution, as well as the traditional extractants, i.e., acid extractant HAc, chelat extractant EDTA and neutral extractant CaCl_2_ [4,5,6]. All of these methods, as the ex situ approaches, were widely applied due to their cheapness and simplicity [3,4,5,6]. However, many investigations indicated that not all of the Cd fractions have toxicity to biota; its bioavailability and mobility depend on its specific forms of fractions [6,7]. Previous research had traditionally divided cadmium into three categories: (1) effective form; (2) potential effective form; and, (3) inert form [6,7,8]. Although the different components could interconvert into each other in certain conditions, the bioavailability of Cd in the ion phase which belongs to the effective form is 20-fold higher compared to the Cd in the complex phase that belongs to the potential effective form [9,10,11,12,13]. Consequently, Zhang et al. [14] revealed that the ex situ measurement always neglected the process of plant uptake. All these approaches were static measurements and sabotaged the original growing environment of plants [15]. These methods involved just a simple step which extracted the metal fractions by applying different intensity reagents; the extracted fractions do not have an inevitable immanent connection with the bioavailable components. Previous investigations have indicated that the physicochemical parameters, i.e., redox potentials variation and the pH variation, could significantly influence the fate and the labile of the Cd fractions in the soil ecosystem [15,16,17]. Consequently, optimizing the in situ, dynamic method to simulate the plant uptake for soil Cd bioavailability monitoring is of significant importance.

The diffusive gradients in the thin film (DGT) technique, as the passive sampling technique, have drawn the attention of many researchers who have seen this technique as a determining mechanism since its creation [16,17]. Based on the Fick’s first law, DGT monitored the diffusion of the dissolved species of Cd through adding a membrane-diffusive layer, which could also control and restrict the flux accumulated in an ion-exchange resin [14,16]. The membrane filter used to protect the inner diffusive gel and resin gel builds a federation, and such a joint federation could form the diffusive layer. The Cd concentration measured by DGT depends on the thickness of the diffusive layer (Δg, 0.93 cm), its diffusion coefficient of Cd^2+^ (D, the value obtained from the http://www.dgtresearch.com/) in the diffusive layer, the exposure window area (A, 3.14 cm^2^), the deployment time (t, 86,400 s) and the accumulated mass of Cd^2+^ over the deployment time (M, ng) [15,16,17].

(1) $$C_{DGT}=\;\frac{M\Delta g}{DAt}$$

The *C*_DGT_-obtained Cd fractions not only included the dissolved amount in the soil concentration, but also contained the components diffused from the further solution and the fractions released from the solid phase; all these components constituted the labile fractions of the biota. Consequently, the *C*_DGT_ could be considered as a chemical surrogate for the biota. Moreover, it could eliminate the accidentalia because its measured concentration was the mean concentration of elements during the deployment time. In view of the distinct feature of the dynamic process, the DGT-measured concentration can truly reflect the bioavailability of elements because of the different ingredient of the resin gel. The related study showed that the AG50W-X cation-exchange resin gel could be applied for Cs and Sr evaluation [18]; silver iodide distributed in the resin gel could adsorb sulphide fractions [19]; the resin gel containing ferrihydrite could monitor the labile phosphorus [17]; the resin with thiol groups could fix the flux of Hg [20,21,22]; and the resin with common chelex-100 particles could be applied to predict the bioavailability of bivalent and trivalent metal ions toxicity [23].

There existed good correlations between the DGT-measured concentrations and the accumulated concentrations in plant tissues; however, researchers have not reached a consensus on the good performance of the DGT technique. A number of researchers contend that DGT-measured concentrations of Cd in soils were not well correlated with Cd concentrations in wheat [24], lettuce [25] and ryegrass [26] and the previous experimental results indicated that the DGT was not a robust tool for evaluating the Cd bioavailability; its measured labile concentrations were easily influenced by the species of accumulated biota and the physicochemical properties of soils [27,28,29]. As we know, different growth periods accounted for the different rate of uptake, but the DGT mimic the uptake processes mechanically. The optimized results achieved by DGT probably change according to the mean rate of biota uptake. Therefore, further investigation of the DGT for Cd bioavailability in the extreme soil ecosystem such as Yixing is essential to environmental research.

Five traditional ex situ methods including soil solution concentration, microwave digestion Cd concentration and three chemical extraction methods (chelating extractant-EDTA, acid extractant-HAc and salt solutions-CaCl_2_), as well as the in situ technique of DGT, were applied as the Cd evaluation tool in this study. The typical cash crops in Yixing—paddy and zizania aquatica—were supplied for the selection of the plant with tolerance to labile Cd fractions. Linear correlations between the different indicator-measured Cd concentrations and plant-accumulated Cd concentrations were used to identify the best-suited indicator for Cd evaluation in this study. Moreover, the multiple regression models were also applied to select the ideal method, which is independent of soil physicochemical properties’ variation, for Cd bioavailability evaluation. The objective of this study is to select the suitable cash crop with high tolerance to Cd contamination. Furthermore, we aim to provide an ideal and robust tool for Cd contamination evaluation in Yixing.

## 2. Experimental

### 2.1. Soil Sites

**Figure 1:**
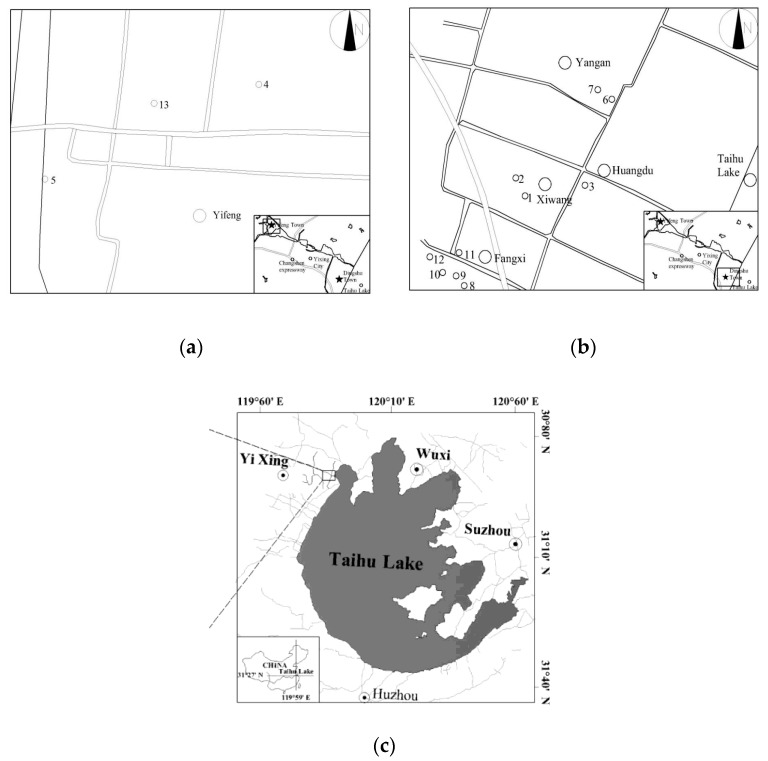
The distribution of sampling sites, (**a**), (**b**) were the distribution of the sampling sites and the (**c**) was the located of the Yixing.

Yixing is a city famous for its special Zisha ceramic products. However, due to the anthropogenic impact on the soil environment, the raw material of Zisha might be significantly affected by Cd pollution. To gain an overall understanding the Cd contamination, samples were collected from 12 sites in Yixing, Jiangsu China (Figure 1). Sampling sites 1 and 2 were located in Yifeng town, where the soil ecosystem might have little influence of Cd contamination. Consequently, sampling sites 1 and 2 were selected as the control group. The other sampling sites were located in the town of Dingshu. Sampling sites 3 and 4 were located in the Yangan, which has many non-ferrous metal smelters. Sampling sites 5, 6 and 7 were the field for the paddy-grown soil. Sampling sites 8–12 are located in the Fangxi, where there are many pottery workshops. Moreover, sampling sites 11 and 12 were the fields where zizania aquatica was grown. The collected soil samples were frozen under −80 °C in the ultra-low temperature freezer, then freeze-dried by the vacuum freeze dryer (B16-333-8811, LABCONCO Ltd., Kansas, USA), and finally sieved with 2 mm stainless steel mesh. Prior to the pot experiment, the total Cd for each crop was digested by the commonly used aqua regia method and determined by flame atomic absorption spectrophotometry (Z-81001, Hitachi Ltd., Hitachi, Japan) with the method detection limit (0.002 mg·L^−1^ Cd). Quality control of the analytical method was conducted every ten samples using a certified standard solution to ensure accuracy and precision with experimental errors (<5%). The physiochemical properties of the samples are showed in Table 1.

### 2.2. Greenhouse Pot Experiment

To ensure the repeatability and accuracy for Cd evaluation, two typical cash crops—paddy and zizania aquatica—were selected as the supplied plants. The fifteen seeds of paddy and six seeds of zizania aquatica were sown in the pots filled with 0.75 kg of the collected soils and three replicate tests were carried out for every soil site, and 55% and 75% maximum moisture content were kept in paddy- and zizania aquatica-grown pots, respectively. After germination, in order to keep an equal number of seedlings per pot, we reduced the number of seedlings to 10 paddy and four zizania aquatica seeds, respectively. All plants were under the natural day–night cycle and grown with no nutrient addition in a greenhouse. During the plant growth process, deionized water was added every day to maintain the soil moisture to reach the 55% and 75% maximum moisture content for paddy- and zizania aquatica-grown soil, respectively. Given that the growth of zizania aquatica requires large quantities of water, the pots with the zizania aquatica-grown soil were hydrated twice a day. After four weeks of growth, smut fungus was applied to stimulate the fresh stem of zizania aquatica. After eight weeks of growth, all plants were harvested and separated into shoots and roots. The collected shoots of the plants were rinsed with tap water and further cleaned with deionized water in order to sweep away the fine particles adsorbed on the root surface. Then, the roots were soaked in the 20 mmol∙L^−1^ EDTA for 15 min and then washed with deionized water; all these procedures were to exclude the interference of Cd fractions adsorbed onto the root surface. The shoots and roots of the plants were dried in an oven at 70 °C for 4 h to wipe off chlorophyll, after which the temperature was reduced to 50 °C to a constant weight. Dry weights were successively recorded. Cadmium concentrations in plant tissues were determined by flame atomic absorption spectrophotometry (Hitachi Z-81001, Hitachi Ltd., Hitachi, Japan) with the method detection limit (0.002 mg·L^−1^ Cd). To ensure accuracy and precision with experimental errors (<5%), quality control of the analytical method was conducted on ten samples at a time using a certified standard solution. After harvest, the remaining soils were air-dried at room temperature and sieved with 2 mm stainless steel mesh for the following analysis of various parameters.

### 2.3. Analytical Methods for Cd Evaluation

#### 2.3.1. Ex Situ Measurement

*Single extraction methods*: Three widely used single extraction methods were selected to measure the bioavailable Cd fractions in soil pools because of their different characters and extracted intensity. The extractants were 0.11 mol·L^−1^ HAc [27,29], 0.05 mol·L^−1^ EDTA and 0.01 mol·L^−1^ CaCl_2_ [28,30]. Among the three methods, the 0.11 mol·L^−1^ HAc was the first step in a three-step sequential extraction procedure recommended by the European Community Bureau of Reference (BCR) [27,31,32,33]. EDTA is a commonly used chelating agent with a strong bonding force, which can complex with most metal ions. CaCl_2_ is a typical neutral agent, which, based on the principle of ion exchange, was used to measure the target elements.

All extraction procedures were conducted in triplicate. The extracted solutions were centrifuged at 3000× *g* for 20 min at 25 °C (avoid the extracted process overheating due to the high-speed rotation). The supernatants were filtered with the strainer and then transferred to leach solution in 10 mL centrifuge tubes. The solution was acidified with nitric acid and stored in a refrigerator at 4 °C prior to analysis. The detailed operations of the three main procedures for metals extraction are listed in Table 2. The procedure just described is shown as the first listed method (HAc^1^).

*Total Cd concentration in soil*: The total Cd concentration in soil was determined by the microwave digestion of aqua regia according to the published methods [29,30,31]. The total amount of Cd in soil solutions was measured using atomic absorption spectrophotometry (Hitachi Z-81001, Hitachi Ltd., Hitachi, Japan).

*The dissolved Cd concentration in soil solution*: The concentrations of Cd in soil solutions (*C*_sol_) were measured according to the traditional centrifugation method. The soil solution was obtained at the 80% maximum moisture content, and the soil solution was collected by centrifuging (10,000× *g*) the paste soils for 20 min at 25 °C. To ensure the purity of the analytes, the supernatants were filtered through a 0.45-μm pore size cellulose nitrate filter membrane and then were acidified by nitric acid [31,34,35]. The Cd concentrations in soil solutions were measured using atomic absorption spectrophotometry (Hitachi Z-81001, Hitachi Ltd., Hitachi, Japan).

#### 2.3.2. In Situ Measurement

**Figure 2:**
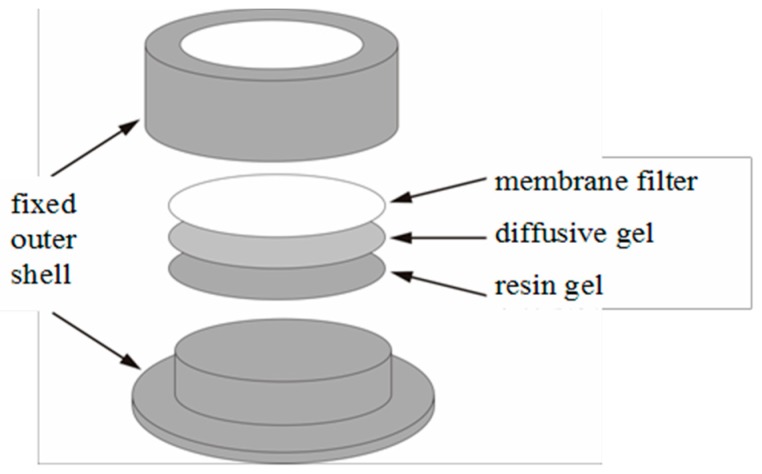
Schematic view of a diffusive gradients in thin film (DGT) device.

*DGT technique*: Figure 2 is the schematic view of a DGT device applied in the soil. The piston-type DGT device assembled from bottom to top consisted of a plastic base, a resin gel, a diffusive gel, a protective membrane filter and a plastic cap. The exposure window with the determined soils is 2 cm in diameter (the plastic base and cap were purchased from DGT Research Limited, Manchester, UK). The diffusive gel (0.8 mm thickness) was made by adding 15% acrylamide and 0.3% agarose-derived cross-linker following a published procedure [15,16,17,34]. The resin gel (0.4 mm thickness) was made by inserting Chelex-100 into diffusive gel. In order to protect the inner gel, in the DGT assembly, a 0.13 mm cellulose-nitrate filter membrane (0.45-µm pore size; Whatman, Maidstone, UK) was placed above the resin gel [14]. The application of DGT in soils could be simply divided into three steps as follows [36]:

*Pretreatment of the soil subsample*: Firstly, the maximum water holding capacity (MWHC) of the soil subsample was measured under specified temperature conditions. The subsample was then placed in a 100 mL plastic pot while, at the same time, the soil was kept at 60% MWHC using deionized water under a fixed saturation for 48 h. Then, moisture content was modulated up to 80% MWHC for 24 h before DGT deployment.

**Figure 3:**
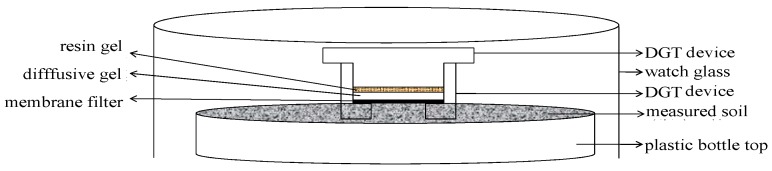
Schematic view of DGT deployment in soil.

*Deployment of DGT device*: To ensure complete diffusion between the soil paste and DGT device, the top of the DGT was gently pressed to guarantee the touch area of the DGT device. The assembled DGT devices were deployed for 24 h at 25 ± 1 °C during the deployment of the DGT, and the temperature and moisture content were controlled at the constant state (Figure 3).

*Retrieval and elution of DGT*: Filter membranes were given 24 h to accumulate soil particles, which were removed by deionized water. After carefully disassembling the device, resin gels were transferred into a micro vial filled with 1 mL of 1 mol·L^−1^ nitric acid for at least 16 h. The concentrations of cadmium in the eluent were measured by atomic absorption spectrophotometry (Hitachi Z-81001).

### 2.4. Data Analyses

The relationships between various bioavailable indicators of Cd measured by the chosen methods and the concentration of Cd in plant tissues were investigated using Word. Statistical analyses were performed using the SPSS statistical package (version 10.0 for Windows, IBM, New York, NY, USA). The multiple regression models were established to describe the performance of the indicators.

## 3. Results and Discussion

### 3.1. Plant Growth

**Figure 4:**
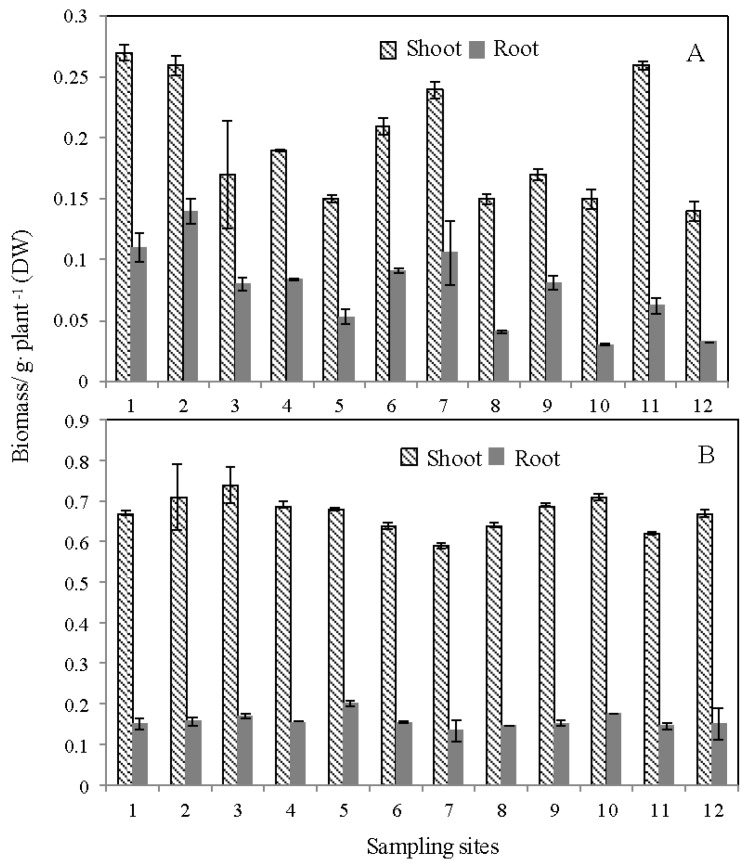
The biomass of paddy (**A**); and zizania (**B**) in the 12 sampling sites of Yixing.

As Figure 4 showed, the paddy and zizania aquatica seemed to have significant differences in their biomass. The biomass of paddy has an extremely obvious variation in the different sampling sites. However, the biomass of zizania aquatica seemed not to be influenced by the Cd contamination concentration. The zizania aquatica, as the widely cultivated vegetable, has the stringent requirement on the water quantity compared to the paddy. Consequently, in theory, the zizania aquatica might show inhibited performance in biomass due to more leaching mass of the labile Cd fractions compared to the paddy. The zizania aquatica indicated that the tolerance to Cd contamination might be due to its extremely developed root and abnormal growth stem. The zizania aquatica biomass of roots showed significant superiority for shoots during generation and the seedling period, but its fresh stem will be enlarged by infected smut fungus at the last phase of the seedling period. All these phenomena arise in the extremely large biomass of zizania aquatica shoots compared to zizania aquatica roots, as well as the paddy shoots and roots. However, the investigation by Yao et al. (2015) indicated that the biomass of typical terrestrial plants, wheat and maize were significantly restrained by the additional Cd in the Cd-contaminated soil (Figure S1 in the Supplementary Information) [37]. The Cd fractions applied in the simulation experiment showed stronger bioavailability in the biota compared to that in Yixing. Due to the strong aging effect on the Cd-contaminated soil in the simulation experiment, the role of the biota auxin and the soil organisms and microorganisms, which leeched into biota growth, merited our attention [17,35]. Further investigation will support this speculation.

**Figure 5:**
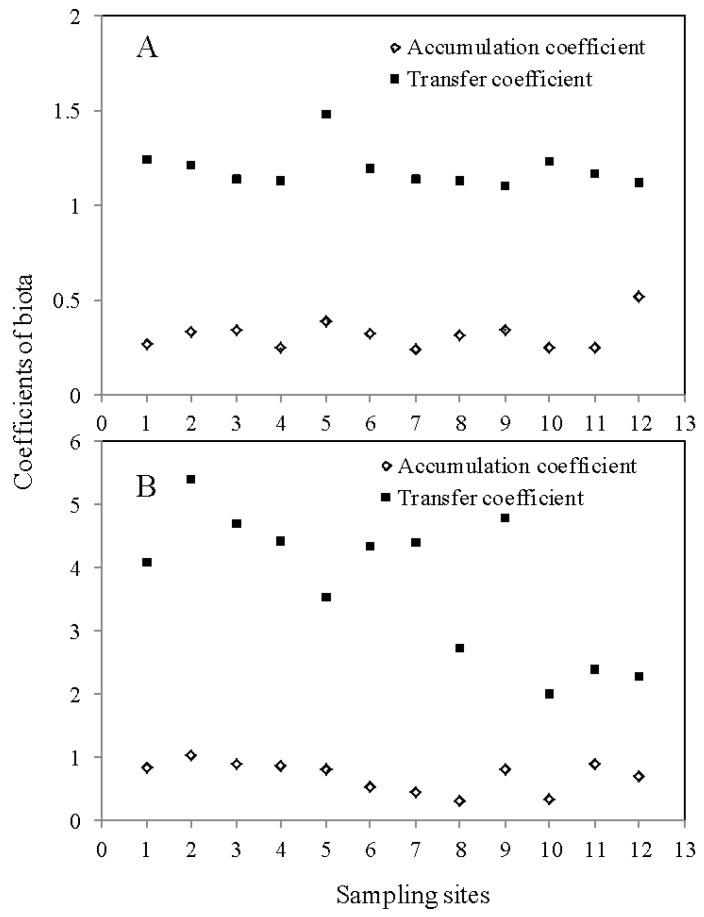
The accumulation coefficients and transfer coefficients of paddy (**A**); and zizania (**B**).

To demonstrate the accumulation effects of the zizania aquatica on the Cd-contaminated soil, the accumulation coefficient (the ratio of plant-accumulated Cd concentration to soil total Cd concentration) and the transfer coefficient (the ratio of shoot-accumulated Cd concentration to root-accumulated Cd concentration) of paddy and zizania aquatica were exhibited in Figure 5. The accumulation coefficients of paddy and zizania aquatica varied from 0.23 to 0.52, and 0.31 to 1.02, respectively. This phenomenon demonstrated that zizania could effectively accumulate the Cd fractions and reduce the total Cd concentrations in soil. Furthermore, the zizania aquatica tend to transport the Cd fractions upward from paddy, which is indicated by the zizania aquatica transfer coefficients from 2 to 5.38, compared to that of paddy from 1.1 to 1.47. The zizania aquatica showed an extremely outstanding performance in Cd contamination improvement. However, due to the extremely high transfer coefficients of the zizania aquatica, the zizania aquatica stem, as the typical edible cash crop, might have a potential risk to human health through bioaccumulation and biomagnification. Consequently, the zizania aquatica could not be selected as the accumulation biota for Cd-contamination improvement. Moreover, the zizania aquatica is not recommended as the main cash crop in Yixing.

### 3.2. Different Indictors for Cd Bioavailability Evaluation

**Figure 6:**
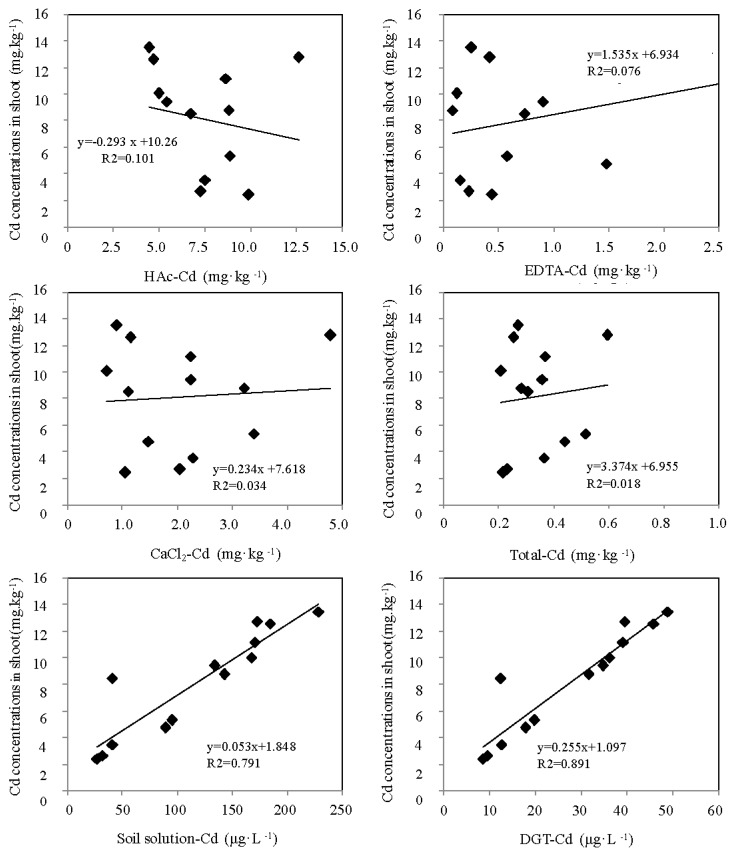
Correlation between Cd concentrations in plant tissues and bioavailable concentrations of Cd measured by six methods in paddy-grown soils.

**Figure 7:**
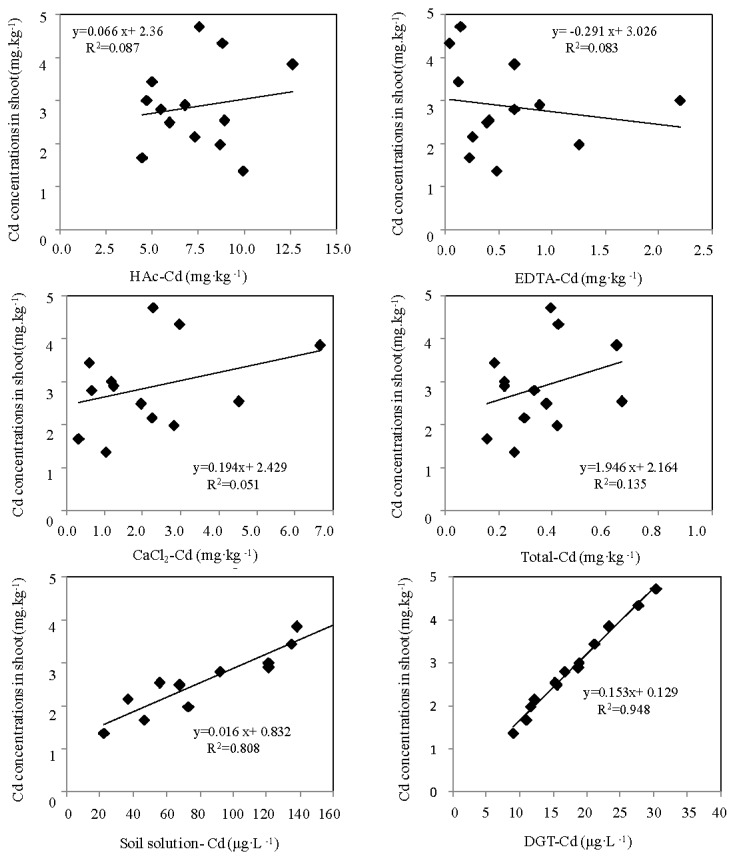
Correlation between Cd concentrations in plant tissues and bioavailable concentrations of Cd measured by six methods in zizania-grown soils.

The shoot was selected for Cd accumulation because the shoots were easier to collect compared to the roots. Furthermore, since the shoot was the edible part of paddy and zizania aquatica, it might have a direct relation with human health. The relationship between the different indicator-measured Cd concentration and Cd accumulation concentration in the shoot of the paddy and zizania aquatica were shown in Figure 6 and Figure 7, respectively. The DGT-measured Cd concentration and the total Cd concentration in soil solution showed a significant linear correlation with the accumulated Cd concentration in plant shoots. However, the EDTA, HAc and CaCl_2_ measured Cd, as well as the total Cd concentration in soil, seemed to have no relationships with the Cd accumulation concentration in plant tissues.

It has been recognized that the DGT technique only measured labile species, and excluded kinetically inert organic species, large colloids and strong organic-metal complexes [25,26,38]. As a dynamic, biota simulation tool, it could obtain Cd concentration including the fractions in pore water and those related to the dynamic resupply of elements from complexes in soil solutions and solid phases [26,39]. Consequently, DGT can be used as an ideal tool for Cd bioavailability evaluation in soils. The soil solution also showed obvious advantages compared with the other ex situ measurements. Due to the fact that plant roots directly take up mineral elements from the soil solution, the concentration of Cd in soil solution was considered a good indicator for plant availability prediction [25,26,27,39,40]. Previous studies showed that total dissolved Cd in soil solutions is a more direct means of estimating the potential for Cd uptake by plants [26,41,42,43]. Similar studies have reported that the concentration of Pb, Zn and Ca in soil solution could reflect the bioavailable fractions for plants uptake [41,42,43,44]. Accordingly, the Cd concentration in soil solution as a typical ex situ approach might be feasible to evaluate the bioavailable Cd fraction in soil.

In this study, given the poor performance of the single extraction methods for the ex situ measurements, they could not reflect the bioavailable level of Cd. Among them, EDTA, as a chelating agent, extracted the largest amounts of Cd from soils compared with the other two extractants (HAc and CaCl_2_); it extracted target elements including the organically bounded fractions and components in oxides or secondary clay minerals [26,40]. HAc indicated a larger Cd extraction ability than CaCl_2_; HAc-extracted Cd was the mixture of organic matter-bounded Cd and calcium carbonate/minerals-bounded Cd. CaCl_2_ as the neutral extractants, based on the ion-exchange principle, measured the target elements, but the exchange capacity was limited by the concentration of the Ca^2+^, and it was easily affected by the soil texture [41,45,46,47,48,49]. We came to the conclusion that single extraction methods have laid the foundation for analysis error, due to their extraction intensity and extraction time; moreover, the concentration of the extractant restrained the empirical value, and led to the mismatching of the labile component in the soil. In addition, the static extraction could not establish an equal relationship with the dynamic uptake process. Consequently, all these deficiencies hindered the accuracy analysis of ex situ methods for Cd bioavailability evaluation.

The ex situ technique-measured Cd indicated a significant correlation with the accumulated Cd concentration in the plant tissue in previous simulation experiments (Table S1 in the Supplementary Information) [37]. Excluding the ageing effect, the supplied biota, paddy and zizania aquatica might result in the poor performance of the ex situ measurement. Accordingly, the traditional ex situ measurement has non-ideal performance in Cd-contaminated soil, which has the cultivation process of the Cd tolerance biota.

### 3.3. Multivariate Analysis

Upon further analysis of the pros and cons of the selected methods, multiple regression models were applied for the optimization [49,50]. Physicochemical properties which might affect the process of plant uptake in both the solid phase and solution, including pH, OM, CEC, and soil texture were selected as the index for analysis. In order to simplify the soil properties from multidimensional to lower-dimensional parameters, principal components analysis (PCA) was used before establishing the multiple regression models. Regarding the mechanical composition of the soil, with the particle size of clay, it has economic value for Zisha products. In addition, compared to other particle sizes, the clay soil particles were effectively proportioned to bind to metals. Consequently, the silt and sand proportion were neglected in PCA.

Firstly, the variance of the data set with interrelated variables (pH, DOC, CEC, clay proportion, the concentration of the TP, TN and K as well as the labile Zn and Pb concentrations in the soil ecosystem) was classified to the independent variables which were called principal components (PC); then, taking the eigenvalues >1 as the extraction criterion, two PCs were extracted. The first (PC1) and second PC (PC2) accounted for 69% and 27%, respectively, and this simplification could explain 90% of the total variance of the analysis data. The factors of PC1 for CEC, OM and clay proportion were 0.811, 0.675 and 0.524; the factors of PC2 for pH, TP and labile Zn were 0.414, 0.219, and 0.792. The CEC, OM and clay proportion were obviously correlated with the PC1 which was representative of the “organic matter”; likewise, the pH, TP and labile Zn which primarily correlated with PC2 were representative of “inorganic matter”. More specifically, the “inorganic matter”, as the competing constituent, interfered in the labile fractions complex of the “organic matter” during the uptake process of the plant, and these mechanisms dictated the bioavailability and liquidity of Cd.

Multiple regression models were built to explain how PCs influence the relationships between various bioavailable indicators and the accumulations of Cd by wheat and maize. The designated bioavailable indicator and the PCs were chosen as the input and the Cd concentration in the plant tissue of wheat or maize was chosen as the output. The performance condition of the DGT technique, soil solution, HAc, CaCl_2_, EDTA and total amount were intuitively reflected through the regressions (Equations (1)–(12)). The results of the evaluation indicated that wheat-grown and maize-grown soil of DGT were significantly influenced by PC1 and PC2 (Equations (1) and (7)); the traditional chemical methods were only influenced by PC2 (Equations (2)–(6) and (7)–(12)). The investigated results showed that the correlation coefficients of paddy-grown soil obtained by HAc, CaCl_2_, EDTA, soil solution and total amount concentration were from 0.101, 0.034, 0.076, 0.791 and 0.018 and rose to 0.51, 0.46, 0.42, 0.83 and 0.56, respectively; the zizania aquatica-grown soil obtained by HAc, CaCl_2_, EDTA, soil solution and total amount concentration were from 0.087, 0.051, 0.083, 0.808 and 0.135 and rose to 0.33, 0.45, 0.42, 0.86, and 0.59, respectively. The intuitive understanding of the numerical amounts, with the aid of the models, took the principal impacts of plants’ uptake into account, so the multiple regressions were considered to embrace more variance compared with the simple regression. The results indicated that the correlation coefficients of HAc, CaCl_2_, EDTA, soil solution and total amount concentration of paddy-grown and zizania aquatica-grown soil all showed marked increases. Taking the complex physicochemical properties into account, the phenomenon of the great improvement in the correlation coefficients between the traditional ex situ Cd bioavailability indicators and accumulated Cd in biota shoot might be the result of the weakened disturbance factors. The ex situ approaches could not reflect the influence of the relevant properties in the soil ecosystem; they are just the embodiment of the equilibrium state of the components of target elements. In addition, the multiple regression models could eliminate the disadvantages of the ex situ measurements, and simplify and optimize the correlation between the indicators and bioaccumulation. However, the DGT-measured Cd did not seem to be optimized after the multiple regression models. The mobile Cd fractions transferred from the soil surface to pore water, through the DGT membrane filter and diffusive gel, then bound to the resin gel during the DGT device accumulation. Meanwhile, the physicochemical properties relevant to this dynamic transfer process, which occur during uptake were embraced by the DGT accumulation. Accordingly, the DGT technique was the simulation process of plant uptake. Consequently, the values after the optimization by the multiple regression models for DGT show no excellent performance; this phenomenon also demonstrates that the DGT technique was a robust tool for Cd bioavailability evaluation in the Cd-contaminated soil of Yixing.

paddy-Cd = 0.13DGT + 0.353(I) + 0.167(II) + 0.83 R^2^ = 0.89** (1)

paddy-Cd = 0.71HAc − 0.638(II) + 2.723 R^2^ = 0.51* (2)

paddy-Cd = 0.241EDTA − 0.701(II)+2.42 R^2^ = 0.46* (3)

paddy-Cd = 2. 12CaCl2 − 0.615(II) + 1.55 R^2^ = 0.42* (4)

paddy-Cd = 0.22total − 0.847(II) + 0.985 R^2^ = 0.56* (5)

paddy-Cd = 0.1soil solution − 0.39(II) + 1.416 R^2^ = 0.83** (6)

zizania-Cd = 0.037DGT + 0.33(I) + 0.723(II) + 0.158 R^2^ = 0.93** (7)

zizania-Cd = 0.665HAc − 1.456(II) + 7.857 R^2^ = 0.33* (8)

zizania-Cd = 10.072 − 0.843EDTA − 1.95(II) R^2^ = 0.41* (9)

zizania-Cd = 16.83CaCl2 − 2.818(II) + 14.26 R^2^ = 0.45* (10)

zizania-Cd = 16.243 − 1.154total − 2.18(II) R^2^ = 0.59* (11)

zizania-Cd = 0.068soil solution + 0.589(II) + 0.56 R^2^ = 0.86** (12)

The previous investigation also revealed the advantages of DGT for Cd bioavailability evaluation. Nolan et al. [11], Zhang et al. [14] and Tian et al. [50] have demonstrated the good performance of Cd on Lepidium sativum; Zn on wheat; and Cu on Lepidium heterophyllum of DGT. They indicated that DGT could fully dynamically reflect the actual condition as well as the resupply intensity of metals attached on the solid surface, which then diffused to pore water. Furthermore, all these advantages were precisely what traditional ex situ measurements could not achieve. Compared with other in situ measurement techniques, the technique of DGT was also shown to be superior in measuring Cu bioavailability in an evaluation of barley [17]. Luo et al. [47] also found that DGT predicted Cd toxicity better than either free Cu^2+^ activity or soil solution. However, as the DGT technique could not embrace all the processes of plant growth and neglected the rhizospheric microorganism, we thought that the DGT technique may sometimes fail to accommodate all the factors that influence plant uptake [24,26]. Further investigation will demonstrate the performance of DGT for different growth periods.

## 4. Conclusions

This study showed that the typical local cash crop, zizania aquatica, was tolerant to and could effectively reduce the total Cd concentration, which was indicated by the high accumulation coefficients. However, owing to its extremely high transfer coefficient, it could not be selected as the accumulation biota for Cd-contamination improvement. Moreover, the zizania aquatica, as the main cash crop in Yixing, might be an unreasonable agricultural structure.

Furthermore, the DGT technique showed better performance for the Cd bioavailability prediction in paddy and zizania aquatica soil compared to the typical ex situ methods i.e., soil solution, HAc, EDTA, CaCl_2_ extractants and microwave digestion. The DGT measurement embraced the principle that physicochemical properties components influence the labile Cd fractions, as indicated by the inconspicuous improvement of the correlation coefficient obtained by multiple regression models compared to linear regression models. Consequently, the DGT technique might be a promising and robust tool for Cd bioavailability evaluation in Yixing. As an in situ, dynamic technique, DGT performed excellently for the time-weighted average concentrations obtained through the pre-concentration process, independent of the physicochemical properties. The advantages of the DGT technique were noticeable in this study. However, quantification of the target labile fraction is not straightforward during accumulation by the DGT device. The DGT process was not sufficiently sensitive to reflect the rhizosphere microorganism and the organism’s effects due to the process’s comparatively long deployment time for the target element in a low-contaminated ecosystem. Consequently, the accurate accumulation of DGT for extremely low concentrations of the target elements should be improved.

## Figures and Tables

**Table 1:** The basic physical and chemical properties of sampled soils.

Site	pH	MC (%)	OM (%)	Soil Mechanical Composition			CEC	Zn Centration (mg·kg^−1^)	Pb Centration (mg·kg^−1^)
				Clay	Silt	Sand			
**1**	5.93	35.6	4.31	8.51	47.19	14.42	14.3	103.5	53.98
**2**	6.18	38.1	4.19	13.62	35.27	19.46	16.6	132.4	67.37
**3**	6.75	39.8	5.35	16.47	47.98	14.12	18.7	178.9	94.76
**4**	6.73	41.1	2.49	13.11	49.07	15.01	16.1	141.7	65.07
**5**	6.54	43.4	5.17	12.48	49.94	11.13	15.8	176.9	59.98
**6**	5.78	29.8	4.59	9.85	50.19	14.34	15.3	153.8	71.79
**7**	5.43	31.9	3.39	14.28	44.97	12.92	16.5	201.2	65.34
**8**	5.76	35.7	5.41	9.89	56.18	10.59	16.2	191.5	61.31
**9**	5.94	40.8	2.77	13.78	51.03	13.09	18.8	163.2	71.59
**10**	6.32	41.6	3.19	14.78	52.76	12.91	19.1	167.3	81.35
**11**	7.45	43.2	2.99	17.61	53.15	11.48	21.2	178.9	77.08
**12**	7.27	42.7	4.79	17.11	46.14	16.41	15.7	213.2	61.34

The MC, OM and CEC represent the moisture content, organic matter and cation exchange capacity, respectively.

**Table 2:** The procedures of the three extraction methods adopted in this study.

Extractants	Procedure	References
**HAc**	0.5 g of soil was extracted with 20 ml of 0.11 mol·L^−1^ HAc and shaken for at least 16 h (overnight)	Houba et al. [30,33]
**EDTA**	2.0 g of soil was extracted with 20 mL of 0.05 mol·L^−1^ EDTA adjusted using an ammonia solution to pH = 7.0 and shaken for 2 h	Wear and Evans [31,32]
**CaCl_2_**	2.0 g of soil was extracted with 20 mL of 0.01 mol·L^−1^ CaCl_2_ and shaken for 3 h	Novozamsky et al. [31,33]

## Supplementary Materials

The following are available online at www.mdpi.com/1660-4601/14/3/297/s1, Figure S1: The biomass of wheat (A) and maize (B) grown in soils added with different levels of Cd. Each value is the mean ± SD (standard deviation, n = 3), Table S1: Linear correlation coefficients (r) between Cd concentrations in the plant tissues and bioavailable concentrations of Cd measured by eight methods in soils.
