# Supplementary material for: The Evaluation on the Cadmium Net Concentration for Soil Ecosystems
Source: Int J Environ Res Public Health. 2017 Mar 12;14(3):297. doi: 10.3390/ijerph14030297 (PMC5369133; doi:10.3390/ijerph14030297)
Supplement: Supplementary file 1 [file ijerph-14-00297-s001.pdf]

*Int. J. Environ. Res. Public Health* **2017**, *14*, 297; doi:10.3390/ijerph14030297 

# Supplementary Materials: The Evaluation on the Cadmium Net Concentration for Soil Ecosystems

**Yu Yao, Pei-Fang Wang *, Chao Wang, Jun Hou and Ling-Zhan Miao**

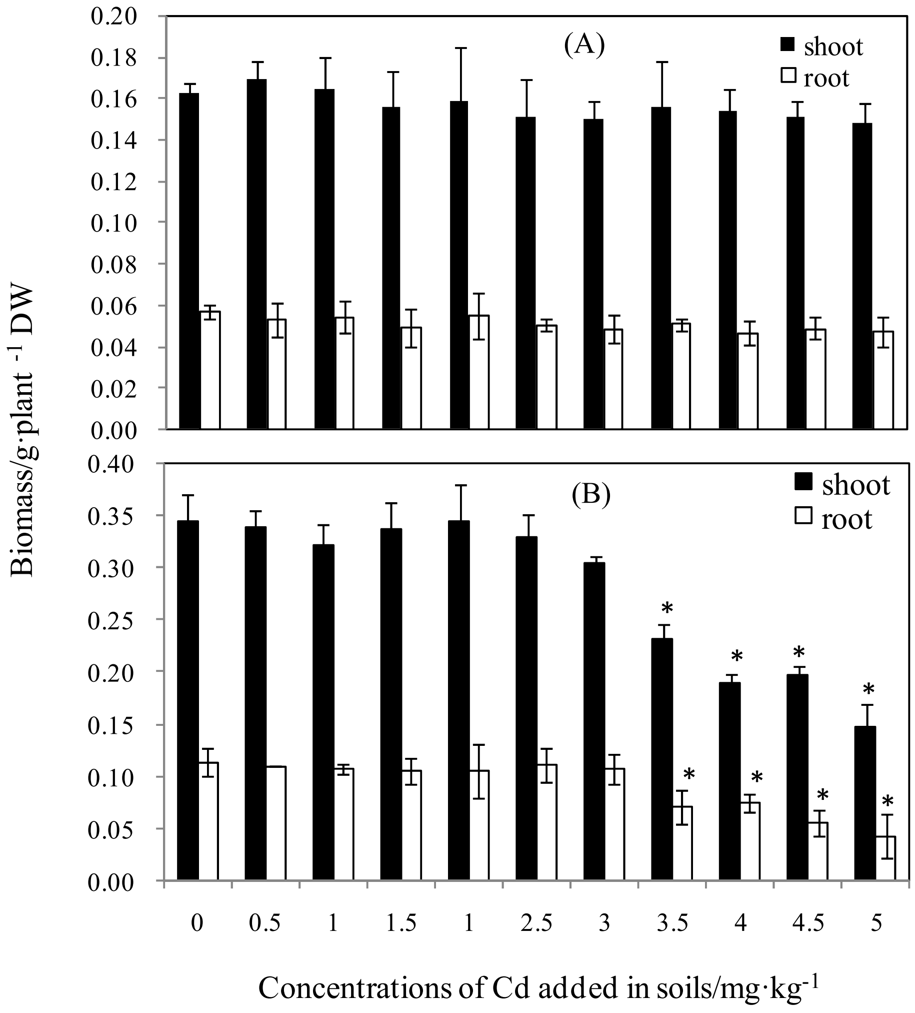


**Figure S1.** The biomass of wheat (A) and maize (B) grown in soils added with different levels of Cd. Each value is the mean ± SD (standard deviation, n = 3).

**Table S1.** Linear correlation coefficients (r) between Cd concentrations in the plant tissues and bioavailable concentrations of Cd measured by eight methods in soils.

| Plant species | Plant tissues | $C_{DGT}$ [a] | $C_{sol}$ [b] | HAc [c] | EDTA | NaAc | $NH_4Ac$ | $MgCl_2$ [d] | $CaCl_2$ |
|---|---|---|---|---|---|---|---|---|---|
| wheat | shoot | 0.985 ** | 0.969 ** | 0.946 ** | 0.970 ** | 0.939 ** | 0.936 ** | 0.968 ** | 0.937 ** |
| | root | 0.988 ** | 0.986 ** | 0.974 ** | 0.983 ** | 0.968 ** | 0.968 ** | 0.986 ** | 0.956 ** |
| maize | shoot | 0.994 ** | 0.997 ** | 0.984 ** | 0.995 ** | 0.982 ** | 0.930 ** | 0.985 ** | 0.981 ** |
| | root | 0.973 ** | 0.968 ** | 0.977 ** | 0.966 ** | 0.985 ** | 0.947 ** | 0.964 ** | 0.971 ** |

[a] $C_{DGT}$: the DGT-measured concentration of Cd. [b] $C_{sol}$: soil solution concentration of Cd. [c] HAc: the first step in the method of the European Community Bureau of Reference (BCR). [d] $MgCl_2$: the first step in the five-step sequential extraction procedure by Tessier et al. (1979). ** Correlation is significant at the 0.01 level.

© 2017 by the authors; licensee MDPI, Basel, Switzerland. This article is an open access article distributed under the terms and conditions of the Creative Commons by Attribution (CC-BY) license (http://creativecommons.org/licenses/by/4.0/).
